# Curated and harmonized gut microbiome 16S rRNA amplicon data from dietary fiber intervention studies in humans

**DOI:** 10.1038/s41597-023-02254-4

**Published:** 2023-06-02

**Authors:** Cynthia I. Rodriguez, Ali Keshavarzian, Bruce R. Hamaker, Feitong Liu, Genelle R. Lunken, Heather Rasmussen, Hongwei Zhou, Julien Tap, Kelly S. Swanson, Maria Ukhanova, Marion Leclerc, Martin Gotteland, Paola Navarrete, Petia Kovatcheva-Datchary, Wendy J. Dahl, Jennifer B. H. Martiny

**Affiliations:** 1 Dept. of Ecology and Evolutionary Biology, University of California, Irvine, USA; 2 Rush Center for Integrated Microbiome and Chronobiology, Rush University Medical Center, Chicago, USA; 3 Whistler Center for Carbohydrate Research and Department of Food Science, Purdue University, West Lafayette, USA; 4 H&H Group, H&H Research, China Research and Innovation Center, Beijing, China; 5 BC Children’s Hospital Research Institute, Vancouver, USA; 6 University of Nebraska-Lincoln, Department of Nutrition and Health Sciences, Lincoln, USA; 7 Microbiome Medicine Center, Department of Laboratory Medicine, Zhujiang Hospital, Southern Medical University, Guangzhou, USA; 8 State Key Laboratory of Organ Failure Research, Southern Medical University, Guangzhou, China; 9 Universite Paris-Saclay, INRAE, MICALIS Institute, Yvette, Jouy-en-Josas France; 10 University of Illinois at Urbana-Champaign, Department of Animal Sciences, Champaign, USA; 11 University of Florida, School of Medicine, Gainesville, USA; 12 Pendulum therapeutics, San Francisco, USA; 13 Department of Nutrition, Faculty of Medicine, University of Chile, Santiago, Chile; 14 Institute of Nutrition and Food Technology (INTA), University of Chile, Santiago, Chile; 15 Laboratory of Microbiology and Probiotics, Institute of Nutrition and Food Technology (INTA), University of Chile, Santiago, Chile; 16 Institute for Molecular Infection Biology, University of Würzburg, Würzburg, Germany; 17 University of Florida, Food Science and Human Nutrition Department, Gainesville, USA

**Keywords:** Bacteria, Microbial communities

## Abstract

Next generation amplicon sequencing has created a plethora of data from human microbiomes. The accessibility to this scientific data and its corresponding metadata is important for its reuse, to allow for new discoveries, verification of published results, and serving as path for reproducibility. Dietary fiber consumption has been associated with a variety of health benefits that are thought to be mediated by gut microbiota. To enable direct comparisons of the response of the gut microbiome to fiber, we obtained 16S rRNA sequencing data and its corresponding metadata from 11 fiber intervention studies for a total of 2,368 samples. We provide curated and pre-processed genetic data and common metadata for comparison across the different studies.

## Background & Summary

Fiber is naturally present in plants, fungi, animals, bacteria, and can also be synthetically made^1,2^. Dietary fibers are carbohydrates that resist digestion by the small intestine and have physiological health benefits to humans^3,4^. High fiber diets show a risk reduction for or amelioration of various illnesses such as constipation, obesity, diabetes, high cholesterol, heart disease, allergies, among others^5–9^. Furthermore, they are associated with improving mineral absorption, insulin responses, gut barrier permeability, immune system defense, production of beneficial metabolites, and inducing changes in the gut microbiome^1,10^. Fiber can modify the gut microbiome by affecting host secretions and transit stool time. It also serves as fermentative substrate for specific microbes and in turn, alters microbial activity more broadly (e.g., through cross-feeding and competition)^11^.

To understand the influence of dietary fiber on the gut microbiota, researchers have performed dietary fiber interventions among both healthy and unhealthy individuals^12^. These studies usually take a fecal sample from a person before and after their dietary change to assess shifts in the composition of the gut microbiome. Currently, the most common approach to assess microbial taxonomic composition is amplicon sequencing of a portion of the universal bacterial 16S ribosomal RNA (rRNA) marker gene^13^ because of the relatively low cost of next generation sequencing and the variety of tools available for bioinformatic processing. However, it still is challenging to access and harmonize such data to compare across studies, especially when its corresponding metadata is missing or hard to decipher^14^.

Motivated by the investigation of fiber-induced shifts in microbiota and the potential for re-analyzing sequencing data, we screened more than 1,500 abstracts and obtained data from 11 fiber intervention studies performed in healthy human subjects, for a total of 2,368 samples from 488 subjects. The purpose of publishing this data descriptor is to provide a detailed description of these valuable datasets, allow others to re-use the data that was carefully curated, and to promote data accessibility. Here, we present 1) the next generation 16S rRNA amplicon sequencing data which have been pre-processed and checked for quality scores, 2) its corresponding metadata which has been harmonized across studies, and 3) the operational taxonomic unit (OTU) tables that contain the number of reads per sample for each taxonomic unit. The sequencing data was primarily produced by Illumina platforms, but also includes 454 and Ion Torrent technologies. All metadata was curated to include similar columns across studies that are clearly defined in the metadata dictionary. The availability of scientific data and its corresponding metadata in comparable and reusable forms will allow researchers to re-analyze and synthesize these data in new ways to better understand the role of fiber in gut health.

## Methods

### Data collection and harmonization

**Fig. 1:**
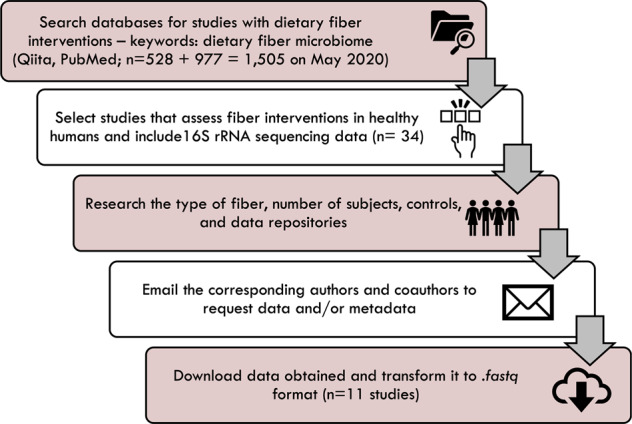
Data collection workflow.

We conducted a keyword search of published literature through the PubMed search engine (keywords: dietary, fiber, and microbiome) under the Best Match algorithm recommended by PubMed on May 9^th^, 2020. The search yielded 977 abstract hits from 2010 to 2020 (https://pubmed.ncbi.nlm.nih.gov/). We also searched through all the records available in the database of open-source microbial management site Qiita^15^ (https://qiita.ucsd.edu) on April 7^th^, 2020 and found 528 microbiome studies including human and animal studies. From both sources, each abstract was carefully read to select studies with fiber interventions in healthy humans that included 16S rRNA amplicon sequencing data from fecal microbial communities (n = 34). We excluded studies in animals and unhealthy humans (Fig. 1). Corresponding authors and first authors were contacted up to 4 times requesting their sequencing data and metadata when not publicly available. We were able to obtain 16S rRNA amplicon sequencing data and their corresponding metadata from 11 studies (Table 1). Data was shared to us via accession number^16–23^ or, if not publicly available, via virtual box. For the studies that did not make their datasets available at the time of publication (Dahl_2016_V1V2, Hooda_2012_V4V6, and Morales_2016_V3V4), we received consent to deposit their data under the BioProject ID: PRJNA891951 to the NCBI Sequence Read Archive^24^. For these studies, we recommend downloading the raw data through the SRA Run Selector Tool that allows users to see the Library Name. Each Library Name includes the study name followed by an underscore and the Sample ID. These Sample IDs are described in the metadata files created for this manuscript (see Data Records and Harmonization of datasets for more information). All studies included in this data repository complied with their relevant ethical regulations and have consent from their human participants to collect and share the data. For more information regarding guidelines for study procedure and trial registration numbers we refer our readers to the individual studies referenced in Table 1 and Table 2. The naming scheme for each of the studies included in this data collection is the following: Last name of the first author in the publication, followed by the year the study was published, and ending with the amplified region of the 16S rRNA bacterial gene (e.g., Liu_2017_V4).

**Table 1:** Data collected and available for eleven fiber intervention studies.

Study Name	Repository for raw data	Accession number for raw data	Sequencing platform used	Single- or paired-end data	Processed data in this manuscript deposited to Figshare repository include
Baxter_2019_V4^35^	NCBI Sequence Read Archive	SRP128128	Illumina MiSeq	paired	cleaned reads, metadata, OTU tables
Dahl_2016_V1V2^36^	NCBI Sequence Read Archive	SRP403421	Illumina MiSeq	paired	cleaned reads, metadata, OTU tables
Deehan_2020_V5V6^2^	NCBI Sequence Read Archive	SRP219296	Illumina MiSeq	paired	cleaned reads, metadata, OTU tables
Healey_2018_V3V4^37^	NCBI Sequence Read Archive	SRP120250	Illumina MiSeq	paired	cleaned reads, metadata, OTU tables
Hooda_2012_V4V6^38^	NCBI Sequence Read Archive	SRP403421	454/Roche pyrosequencing	single	cleaned reads, metadata, OTU tables
Kovatcheva_2015_V1V2^39^	NCBI Sequence Read Archive	SRP062889	454/Roche pyrosequencing	single	cleaned reads, metadata, OTU tables
Liu_2017_V4^40^	European Nucleotide Archive	PRJEB15149	Ion Torrent	single	cleaned reads, metadata, OTU tables
Morales_2016_V3V4^41^	NCBI Sequence Read Archive	SRP403421	Illumina MiSeq	paired	cleaned reads, metadata, OTU tables
Rasmussen_2017_V1V3^42^	NCBI Sequence Read Archive	SRP106361	454/Roche pyrosequencing	single	cleaned reads, metadata, OTU tables
Tap_2015_V3V4^43^	European Nucleotide Archive	PRJEB2165	454/Roche pyrosequencing	single	cleaned reads, metadata, OTU tables
Venkataraman_2016_V4^44^	NCBI Sequence Read Archive	SRP067761	Illumina MiSeq	paired	cleaned reads, metadata, OTU tables

**Table 2:** Summary of data collected by study.

Study Name	Number of interventions	Fibers used in intervention + control when applicable	Amount of fiber or control given in intervention (grams)	Duration of intervention (days)	Collection timepoints	Number of subjects	Number of samples
Baxter_2019_V4^35^	4	Resistant starch from potatoes (RPS), resistant starch from maize (RMS), inulin from chicory root, and an accessible corn starch control	20–40	14	8	175	1,205
Dahl_2016_V1V2^36^	3	RS-4-A, RS-4-B, RS-4-C - Resistant potato starches (RS type 4)	30	14	4	53	212
Deehan_2020_V5V6^2^	4	Tapioca, potato, and maize- Resistant starches (RS type 4) + corn starch control	increments 10–50	28	5	40	200
Healey_2018_V3V4^37^	2	50:50 inulin to fructo-oligosaccharide and maltodextrin control	16	21	4	34	134
Hooda_2012_V4V6^38^	2	Polydextrose and soluble corn fiber control	21	21	3	10	28
Kovatcheva_2015_V1V2^39^	2	Kernel-based bread (BKB) and white-wheat-bread (WWB)	37.6 & 9.1	3	3	20	60
Liu_2017_V4^40^	2	Fructooligosaccharides (FOS) and galactooligosaccharides (GOS)	16	14	4	35	132
Morales_2016_V3V4^41^	2	Oligofructose and maltodextrin control (extra treatments of Orlistat were also given)	16	7	2	41	82
Rasmussen_2017_V1V3^42^	2	Starch-entrapped microspheres and psyllium	9 & 12	84	2	41	82
Tap_2015_V3V4^43^	1	Dietary fiber meals	10 & 40	5	4	19	76
Venkataraman_2016_V4^44^	1	Resistant starch (unmodified potato starch; RS type 2)	48	17	8	20	157

Shows the studies included in this data descriptor and their pertinent information such as fibers used, duration of intervention, number of subjects, etc.

We provide Table 2 with a summary of each of the studies which includes: number of interventions per study, fibers used and their amounts, length of interventions, number of colletion timepoints, subjects and total samples. Because the metadata available was heterogeneous across studies, we performed harmonization across the datasets, so that common variables across studies could be easily identified. The metadata dictionary (Table 3) contains the definition for the data collected across studies.

**Table 3:** Metadata dictionary. Explains each column in the metadata files.

Column Name	Description
sampleid	The name of the fastq file that corresponds to one fecal sample
study	Shows the last name of the first author of the study where the data came from
sample_id_2	Original sampleID depicted in raw sequence reads
subject_id	The ID of the subject (person) that the sample was collected from
treatment	Shows whether the type of treatment administered was a dietary fiber (fiber) or a placebo (control)
timepoint	The time at which this sample was taken - before or after treatment
timepoint_numeric	Defines the time course the fecal sample was taken in chronological order (e.g., 1,2,3..) if coming from the same individual
timepoint_id	Description of the timepoint, including **timepoint** + **timepoint_numeric:** before versus after, with chronological number attached to it, in the case multiple samples were taken from the same individual
sample_name	Has the **subject_id** attached to **timepoint_numeric**
fiber_type	The specific type of fiber that was used in the treatment, and/or the name of the control compound administered
fiber_amount	Grams per day of the compound in the treatment administered, if known (e.g., 20 g/d of inulin)
time_days	The days that had passed since the intervention started, if known. Note that weeks were counted as 7 days, for instance if the intervention lasted 12 weeks, we converted that to 84 days.
number	Order in which samples were originally arranged by the metadata given by authors, should equal number of fecal samples collected
gender	The gender of the subject as reported by original authors (available only for the Healey study)
age	The age in years of the subject reported by original authors (available only for the Healey study)
sample-name-original	The name given to the sample in the original study

To provide as much information on the dietary fiber interventions as possible, we investigated the specific fibers that were used in each study. Table 4 shows all the dietary fibers that were used in the interventions and their manufacturer or recipe (when available) including controls.

**Table 4:** Fibers and placebos given in the interventions.

Fiber type	Description/manufacturer	Study
Resistant starch from potatoes (RPS)	Bob’s Red Mill, Milwaukee, OR	Baxter_2019_V4^35^
Inulin from chicory root	Swanson Health Products, Fargo, ND	Baxter_2019_V4^35^
Hi-Maize 260 resistant corn starch (RMS)	Manufactured by Ingredion Inc., Westchester, IL, and distributed by myworldhut.com	Baxter_2019_V4^35^
Amylase-accessible corn starch (placebo)	Amioca powder; Skidmore Sales and Distribution, West Chester, OH	Baxter_2019_V4^35^
Resistant potato starch RS4-A	PenFibe® RO – 170; phosphorylated, soluble fibre with high viscosity - Penford Food Ingredients Inc., Denver, CO, USA	Dahl_2016_V1V2^36^
Resistant potato starch RS4-B	PenFibe® RO – 177; hydrolysed, phosphorylated, soluble fibre with low viscosity - Penford Food Ingredients Inc., Denver, CO, USA	Dahl_2016_V1V2^36^
Resistant potato starch RS4-C	PenFibe® RS; insoluble fibre with low viscosity - Penford Food Ingredients Inc., Denver, CO, USA	Dahl_2016_V1V2^36^
AMIOCA™ Powder TF (Placebo)	Ingredion Inc, Bridgewater, NJ 08807, USA	Deehan_2020_V5V6^2^
VERSAFIBE™ 2470 (Maize RS4)	Ingredion Inc, Bridgewater, NJ 08807, USA	Deehan_2020_V5V6^2^
VERSAFIBE™ 1490 (Potato RS4)	Ingredion Inc, Bridgewater, NJ 08807, USA	Deehan_2020_V5V6^2^
VERSAFIBE™ 3490 (Tapioca RS4)	Ingredion Inc, Bridgewater, NJ 08807, USA	Deehan_2020_V5V6^2^
Orafti® Synergy1–50:50 inulin to fructo-oligosaccharide mix	Beneo GmbH	Healey_2018_V3V4^37^
Glucidex® 29 Premium-digestible maltodextrin; placebo	Roquette Worldwide	Healey_2018_V3V4^37^
Polydextrose	PDX; Litesse II, Danisco	Hooda_2012_V4V6^38^
Soluble corn fiber (placebo)	SCF; PROMITOR, Tate and Lyle Ingredients	Hooda_2012_V4V6^38^
Kernel-based bread (KBB)	NA	Kovatcheva_2015_V1V2^39^
White-wheat-bread (WWB)	NA	Kovatcheva_2015_V1V2^39^
Fructooligosaccharide- FOS (QHT-Purity95%)	Source: Sucrose; Quantum Hi-Tech (China) Biological company, Guangdong, China	Liu_2017_V4^40^
Galactooligosaccharide- GOS (QHT- Purity95%)	Source: lactose; Quantum Hi-Tech (China) Biological company, Guangdong, China	Liu_2017_V4^40^
Maltodextrin (placebo)	NA	Morales_2016_V3V4^41^
Oligofructose	NA	Morales_2016_V3V4^41^
Starch-entrapped microspheres (SM)	A suspension of sodium alginate (2% w/v) and normal corn starch (9% w/v) was made in water through a special recipe	Rasmussen_2017_V1V3^42^
Psyllium	Natural Foods Inc (Toledo, OH)	Rasmussen_2017_V1V3^42^
Dietary fiber meals (different foods)	NA	Tap_2015_V3V4^43^
Raw unmodified potato starch	Bob’s Red Mill, Milwaukie, OR. This potato starch contains approximately 50% resistant starch (type 2) by weight.	Venkataraman_2016_V4^44^

The description of the compound administered during the intervention as described by the original authors, when available.

### Sequencing processing

Individual studies used different methods for sequencing processing and bioinformatic pipelines, and such differences can influence the diversity and composition of microorganisms detected in a sample as well as the variation observed across samples^25^. Thus, to compare the sequences directly across studies, we obtained the raw sequencing reads for each study and then processed them in a similar manner.

**Fig. 2:**
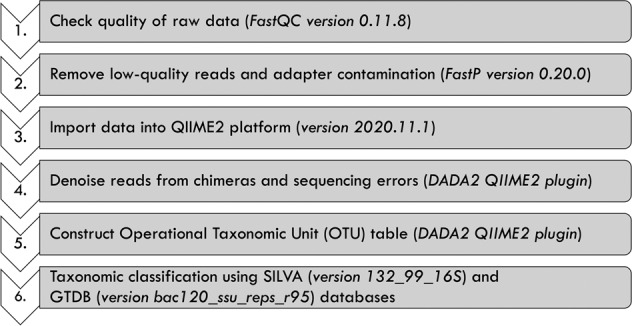
Bioinformatics pipeline for data processing.

First, we assessed the quality of the 16S rRNA sequencing data using FastQC software^26^ (version 0.11.8). The sequencing reads were cleaned from poor quality sequences using the Fastp program^27^ (version 0.20.0). The cleaned sequences were imported into the QIIME2 platform^28^ (version 2020.11.1), and primers were removed using Cutadapt^29^ plugin when necessary. We then denoised the reads using DADA2^30^ plugin, obtaining an OTU table depicting the number of reads per sample for each taxonomic unit (Fig. 2).

Next, the taxonomic classification of the reads was also performed in the QIIME2 platform by training the SILVA^31^ (version 132_99_16S) and the Genome Taxonomy Database^32^ (GTDB; version bac120_ssu_reps_r95) databases to each respective study based on the primers that were originally used (Fig. 2). The SILVA database was used to remove chloroplast and mitochondrial DNA. Then, the cleaned reads were assigned to a final taxonomic group using the GTDB trained database. Reads that were not classified at least to the phylum level were removed from the analysis; sequences were classified to the finest level when possible (e.g., species and/or strain). The sequencing processing and taxonomic classification was performed with both the forward and reverse reads when paired-end data was available. We also repeated the analyses with only the forward reads, and found that both gave very similar results. We provide the OTU tables obtained with both procedures (e.g., baxter_OTU_table_paired_reads.tsv and baxter_OTU_table_forward_reads.tsv) to allow the reader to choose either option for further analysis.

## Data Records

The following data have been deposited in the Figshare^33^ repository: 1) The compressed 16S rRNA sequencing reads (.fastq.gz) containing the amplicon data that were quality filtered as described above; 2) the metadata files per study in tab-delimited format (.txt) describing their corresponding samples serving as a reference to help identify and sort the DNA sequences by different metrics (e.g., timepoint, treatment, individual, etc.); 3) the OTU tables with taxonomic assignment per study (.tsv) presenting the number of reads per sample for each taxonomic unit. As mentioned in the Data collection section and in Table 1, the raw reads for the studies mentioned here can be found in publicly available databases^16–23^. For the studies that did not make their datasets available prior to this publication (Dahl_2016_V1V2, Hooda_2012_V4V6, and Morales_2016_V3V4), we received consent to deposit their data under the BioProject ID: PRJNA891951 to the NCBI Sequence Read Archive^24^.

## Technical Validation

### Data integrity

For quality assurance of the sequencing reads, we utilized the FastQC tool^26^ as it provides quality control statistics such as sequence length, per base quality scores, and adapter contamination^34^. We used the Fastp software^27^ to ensure data integrity: we removed low quality reads from all datasets, only keeping reads with an average quality score of 30, the average score of 25 was chosen in only two occasions (Rasmussen_2017_V1V3 and Liu_2017_V4) because read counts dropped dramatically with a higher threshold (*−average_qual 30 or 25*); we discarded sequences shorter than 100 bp (*−length_required* 100) to remove small sequences that could not complete 16S rRNA amplicon fragments. We only had to remove adapter contamination from one study (Deehan_2020_V5V6) using the detection of adapter correction tool in Fastp (*−detect_adapter*_*for_pe*). When paired-end data was available, we enabled base correction in overlapped regions of paired reads (*−correction*). When corrupted data, having characters that did not belong to the sequencing reads, was found (Hooda_2012_V4V6) we discarded those samples (n = 10).

### Harmonization of datasets

To ensure the datasets were comparable, we converted sequencing reads from all studies into .*fastq* extension files (when necessary). Furthermore, we followed the same pipeline using consistent software and versions (Fig. 2) and cross-validated our results by visually inspecting the sequences after each clean-up step using Geneious prime (version 2020.2.4; https://www.geneious.com). For instance, after removing primers from reads using the Cutadapt plugin in QIIME2, we extracted the reads and imported them into Geneious to verify that sequences had been properly trimmed. Moreover, to ensure clarity and consistency of metadata across datasets, we created a metadata dictionary (Table 3) to explain the data type (categorical, numerical, text, etc.). In most cases, the metadata files available for the studies did not follow a consistent report of variables. For example, there was a big difference in how the timepoints were described (e.g., “before”/“after” vs “post”/“pre” vs numeric) and in most instances the fiber type and grams of fiber were not included. To remedy this, we carefully curated the data collected per sample across studies to have similar naming schemes.

## Data Availability

The parameters and step-by-step scripts used to clean up the data, remove chimeras, and assign taxonomy are available at https://github.com/cirodri1/fiber-data_records (e.g, trimming lengths, primers, databases, etc.).
